# Mate choice for major histocompatibility complex complementarity in a strictly monogamous bird, the grey partridge (*Perdix perdix*)

**DOI:** 10.1186/s12983-017-0194-0

**Published:** 2017-02-16

**Authors:** Dana Rymešová, Tereza Králová, Marta Promerová, Josef Bryja, Oldřich Tomášek, Jana Svobodová, Petr Šmilauer, Miroslav Šálek, Tomáš Albrecht

**Affiliations:** 1 Department of Ecology, Faculty of Environmental Sciences, Czech University of Life Sciences, Kamýcká 1176, 165 21 Prague 6, Czech Republic; 2 Department of Botany and Zoology, Faculty of Science, Masaryk University, Czech Republic, Kotlářská 2, 611 37 Brno, Czech Republic; 3 Institute of Vertebrate Biology, The Czech Academy of Sciences, Květná 8, 603 65 Brno, Czech Republic; 4 Department of Evolutionary Biology, Evolutionary Biology Centre, Uppsala University, Norbyvägen 18D, SE-75236 Uppsala, Sweden; 5 Department of Zoology, Faculty of Science, Charles University in Prague, Viničná 7, 128 44 Prague 2, Czech Republic; 6 Department of Ecosystem Biology, Faculty of Science, University of South Bohemia, Branišovská 1760, 370 05 České Budějovice, Czech Republic

**Keywords:** Grey partridge, Mate choice, MHC genes, Ornaments, Sexual selection, Social monogamy, Inbreeding avoidance

## Abstract

**Background:**

Sexual selection has been hypothesised as favouring mate choice resulting in production of viable offspring with genotypes providing high pathogen resistance. Specific pathogen recognition is mediated by genes of the major histocompatibility complex (MHC) encoding proteins fundamental for adaptive immune response in jawed vertebrates. MHC genes may also play a role in odour-based individual recognition and mate choice, aimed at avoiding inbreeding. MHC genes are known to be involved in mate choice in a number of species, with ‘good genes’ (absolute criteria) and ‘complementary genes’ (self-referential criteria) being used to explain MHC-based mating. Here, we focus on the effect of morphological traits and variation and genetic similarity between individuals in MHC class IIB (MHCIIB) exon 2 on mating in a free-living population of a monogamous bird, the grey partridge.

**Results:**

We found no evidence for absolute mate choice criteria as regards grey partridge MHCIIB genotypes, i.e., number and occurrence of amino acid variants, though red chroma of the spot behind eyes was positively associated with male pairing success. On the other hand, mate choice at MHCIIB was based on relative criteria as females preferentially paired with more dissimilar males having a lower number of shared amino acid variants. This observation supports the ‘inbreeding avoidance’ and ‘complementary genes’ hypotheses.

**Conclusions:**

Our study provides one of the first pieces of evidence for MHC-based mate choice for genetic complementarity in a strictly monogamous bird. The statistical approach employed can be recommended for testing mating preferences in cases where availability of potential mates (recorded with an appropriate method such as radio-tracking) shows considerable temporal variation. Additional genetic analyses using neutral markers may detect whether MHC-based mate choice for complementarity emerges as a by-product of general inbreeding avoidance in grey partridges.

**Electronic supplementary material:**

The online version of this article (doi:10.1186/s12983-017-0194-0) contains supplementary material, which is available to authorized users.

## Background

Sexual selection has attracted the attention of evolutionary biologists since Darwin’s time [1, 2]. In most vertebrate species, females are the choosier sex due to their higher investment in gametes and reproduction, while males are usually exposed to stronger intra-sexual competition for mates [3]. Females can decide upon a male on the basis of direct or indirect benefits [2, 4]. Direct benefits may include such advantages for the female as enhanced fertility or fecundity, avoiding directly transmitted diseases, paternal care, courtship feeding, high-quality territories or better anti-predator defence [2, 4–8]. Indirect benefits may not immediately contribute to the female’s fitness but may enhance offspring viability, resistance to parasites or increase the attractiveness of sons [2, 4, 9–11]. Obviously, these two kinds of benefits are not mutually exclusive (e.g., [12]). While some male qualities may appear to be hidden to females making choices, others may be obvious. Condition-dependent ornaments, olfactory and vocal cues or epigamic displays may help females readily assess a male’s genetic qualities, allowing her to choose the best mate from all available candidates [12–14].

The model of parasite-mediated sexual selection proposed by Hamilton and Zuk [10] assumes that expression of male sexual ornament preferred by females honestly mirrors individual health and vigour, particularly as regards heritable resistance to pathogens and parasites [15, 16]. The major histocompatibility complex (MHC) is a highly polymorphic set of genes encoding transmembrane glycoproteins responsible for pathogen recognition through antigen presentation to T-lymphocytes [12, 17, 18]. MHC genes, therefore, may represent a direct link between mate attractiveness and heritable resistance to pathogens [19]. MHC genes are not only one of the key components of adaptive immunity, they are also involved in mate choice and kin recognition [12, 20]. In some species, individual MHC variants (independently of their function in antigen binding) are detectable by olfactory cues (individual odours), thereby guiding the choice of partner and preventing inbreeding (reviewed in [21]).

Over the past several decades, MHC-based mate choice has been confirmed in a variety of vertebrates, including mammals (reviewed in [20]), reptiles [19], fish and amphibians [22–24] and birds [13, 14, 18, 25–30]. It has become apparent, however, that there is no universal pattern of MHC-based mate choice across species, possibly because of different selection pressures acting on offspring pathogen resistance in different populations. The ‘heterozygote advantage’ hypothesis, for example, assumes that individuals with many different MHC alleles are able to fight more pathogens than individuals with few MHC alleles [12, 31]. If there is no dominant pathogen in a population, therefore, females should choose males with MHC alleles differing from their own using self-referential criteria (‘complementary genes hypothesis’, reviewed in [32, 33]) in order to produce offspring with more MHC variants. In such a case, any benefits to the female are non-additive and arise from the specific combination of maternal and paternal genotypes in the progeny. As individuals with a diverse set of MHC alleles may display a greater risk of autoimmune disorders, however, selection is likely to favour optimal rather than maximal individual MHC diversity [16, 34].

If, on the other hand, one dominant pathogen, or several pathogens with great impact, occur in a population at a given time, then all females should consistently prefer males with a particular allele or genotype providing resistance to those pathogens, thereby displaying mate choice based on absolute criteria (the ‘good genes hypothesis’, [18, 32]). This latter hypothesis predicts additive genetic benefits from choosing high-quality individuals as well as the general attractiveness of bearers of ‘good immune genes’ [35]. Assuming females have no phenotypic cues for recognising presence of a specific allele at a multilocus trait, but can distinguish various levels of male heterozygosity, then an absolute mating preference for the most heterozygous male will be the most advantageous as such mates have a higher probability of carrying the appropriate resistance allele or alleles (the ‘good genes as heterozygosity’ hypothesis [36]).

Reliable phenotypic indicators of male genetic quality are most likely to evolve in lifelong genetically monogamous species as the single mate choice decision will affect lifetime breeding success [12]. An appropriate candidate phenotypic trait for testing female preference according to the good genes hypothesis should ideally be male-specific, heritable, condition-dependent and sufficiently variable among males [37]. Melanins and carotenoids are the two major pigments responsible for most animal colouration [38–40]. Both pigments have been associated with immune function and may act as antioxidants [38, 41]. In the context of mate choice, therefore, melanin- and carotenoid-based secondary ornament in males have frequently been studied as condition-dependent indicators potentially associated with particular MHC alleles or allelic diversity [42].

In birds, the role of MHC genes in mate choice has been documented primarily in polygamous or promiscuous species, while research on monogamous species remains scarce (cf. [29]). Here, we study female mate-choice mechanisms in a free-living population of the grey partridge, a socially and genetically monogamous galliform bird species [43]. The grey partridge is a suitable model species for studying MHC-dependent mate choice because its mating system is non-resource based [44] and a surplus of males enables females to choose a partner from multiple candidates [45, 46]. Ideally, partners form lifelong pairs, though high predation rates often result in repeat mate choices [47, 48]. Despite a relatively low level of sexual dimorphism in this species, the melanin-based plumage ornament on the breast (‘horseshoe’) is usually absent or smaller in females [49]. Carotenoid-based ornament in the form of red skin behind the eye (‘red spot’) occurs in both sexes [50].

We investigated the effect of MHC class IIB (exon 2; hereinafter MHCIIB) genotypes on mating success in grey partridges using several male phenotypic traits (body condition, carotenoid- and melanin-based ornamentation) as covariates in the analysis. The following three hypotheses were evaluated: a) females prefer males carrying a particular MHCIIB amino acid variant (hereinafter variant) and/or a certain phenotypic trait (‘good genes hypothesis’); b) females prefer males with the highest number of MHCIIB variants and/or a certain phenotypic trait (‘good genes as heterozygosity hypothesis’); and c) females prefer males with MHCIIB variants differing from their own (‘complementary genes hypothesis’ and ‘inbreeding avoidance’). The grey partridge mating process was investigated using two different approaches: 1) comparison of characteristics in unpaired and paired males, and 2) comparison of pairwise genetic distances or similarities (i.e., sharing of MHCIIB variants) a) between actual pairs and all possible combinations of candidate pairs on the basis of randomisation, or b) using the preferred male’s rank among all accessible males (i.e., unpaired males at the time of actual pair formation, as assessed through radio-tracking). Finally, we evaluated any potential link between MHCIIB genotype and phenotypic traits in the males preferred by females.

## Results

In total, 15 nucleotide variants (alleles, Additional file 1) were found at MHCIIB exon 2 in the studied grey partridge population (Pepe-DAB*01-04, 06-09, 12-18, GenBank accession numbers: KF007890-93, KF007895-98, KF007901, KY040298-302; Pepe-DAB*18 failed to be cloned). Individuals carried from two to four different MHC alleles (*n*_*1*_ = 56 males: two alleles in 10.7%, three alleles in 46.4% and four alleles in 42.9%; *n*_2_ = 47 females: two alleles in 12.8%, three alleles in 48.9% and four alleles in 38.3%). All females with two alleles (*n* = 5) paired with males having more than two alleles, but the occurrence of individuals having two alleles in real pairs was low. Two of fifteen alleles (Pepe‑DAB*12 and Pepe‑DAB*14) encoded an identical amino acid sequence and thereby an identical peptide. The results presented below are based on variation in more biologically informative amino acid variants.

Results from a set of generalised linear models assessing the effect of particular MHC alleles or their products on male pairing status showed that none of the amino acid variants studied affected male pairing success per se (Additional file 2). Moreover, some alleles (e.g., Pepe‑DAB*03 and Pepe‑DAB*18) occurred at low frequencies in the population (Additional files 1 and 3).

Concerning the possible effect of phenotypic traits or number of MHCIIB variants on male pairing status, only the red chroma of carotenoid-based ornament had a significant effect (Table 1). This model explained just 6.93% of variation in the data. The number of amino acid variants carried by individual males did not affect male pairing success (Table 1). Similar results were also obtained by model selection based on the information theoretic approach (Additional file 4). Mean values of measured phenotypic traits for paired and unpaired males are provided in Additional file 5. Redness of carotenoid-based ornament, the only phenotypic trait potentially influencing male pairing success, was not associated with the occurrence of any of the MHCIIB variants studied (Additional file 6).

**Table 1:** Results of the full model (*n* = 41 males) showing effect of scaled body condition, ornamentation and number of MHCIIB amino acid variants on male pairing status

Predictor	Estimate	SE	z value	*p*
Intercept	−0.072	0.345	−0.209	0.835
Scaled body condition	0.034	0.382	0.089	0.929
Horseshoe area	0.251	0.391	0.643	0.520
Mean red spot area	0.492	0.434	1.135	0.257
Red spot chroma	0.856	0.428	2.000	**0.046**
Amino acid variants number	0.022	0.350	0.064	0.949

Significant values are marked in bold (*p* < 0.05). The pairing status was coded as follows: 0 – unpaired, 1 – paired males

Randomisation tests, based on the assumption that each female can choose any male in the population, indicated that the median of amino acid variant similarity in observed actual pairs was lower than in random pairs (*p =* 0.05; Table 2), the median in actual pairs equalling the lower 95% confidence interval limit. Thus, this analysis is supportive of disassortative pairing in grey partridges at MHCIIB loci in relation to amino acid variants sharing. Unlike the similarity measure, the median of genetic distance based on amino acids for actual pairs lay within the 95% confidence interval limits for random pairs (Table 2; Additional file 7, with results for nucleotide sequences).

**Table 2:** Results from randomisation testing of (dis-)assortative mating in the grey partridge based on amino acid variants of MHCIIB

Variable	95% CI min	95% CI max	Median
Amino acid variants sharing (similarity)	0.3333	0.5714	0.3333
Mean amino acid distance	0.1953	0.2215	0.2156

The median for actual pairs was compared with the 95% confidence interval limits for randomly chosen pairs (based on 10 000 permutations and 36 known actual pairs)

Radio-tracking revealed that monogamous pair formation and increased mortality (Additional file 8) rapidly altered the number and identity of available males during the breeding season (including the pairing period), and that most females did not form pairs immediately after release but did so only after several days or even weeks (see Methods). Taking these points into consideration, we devised a statistical model based on the rank of each preferred male relatively to other relevant unpaired candidates, whose list was unique for each female with regard to the date of her pair formation. In agreement with randomisation results, this analysis proved that females chose more dissimilar males on the basis of MHCIIB variants sharing (*p =* 0.019 after Holm adjustment, Table 3; see Additional file 9 for results based on nucleotide sequences) and that females did not prefer more dissimilar males displaying higher mean genetic distance (Table 3; Additional file 9). Results of the model with mean genetic distance indicated that females paired with males of intermediate rank, highly suggestive of random mating (Table 3).

**Table 3:** Results of generalised linear models assessing (dis-)assortative mating in the grey partridge based on MHCIIB variants using the relative rank of actual mate chosen by each female from unpaired candidates (*n* = 32 females, 5 duplications excluded)

Predictor	Estimate	SE	z value	*p*	*p/2*	Holm
Amino acid variants sharing (similarity)	−0.6751	0.2546	−2.652	**0.0123**	**0.0062**	**0.0185**
Mean amino acid distance	−0.1903	0.2084	−0.913	0.3680	0.1840	0.2840

Significant values are marked in bold (*p* < 0.05). The last column contains probability values after Holm correction

## Discussion

In this study of grey partridge mate choice based on MHCIIB exon 2, we obtained good support for the ‘complementary genes’ hypothesis or ‘disassortative mating’, rather than the ‘good genes’ hypothesis. Our data show that pairs were formed between individuals with more dissimilar genotypes at MHCIIB loci (based on shared amino acid variants) than would be expected through random choice, indicating that females use relative criteria when choosing mates. However, we could not confirm the occurrence of (dis-)assortative mating at mean amino acid or nucleotide distances between paired partners. Although the traditional randomisation-based statistical approach was able to discern differences from random mating in similarity of amino acid variants, the rank-based model with precise definition of candidates revealed differences from random mating in both similarity of amino acid variants and nucleotide allele similarity. The fact that amino acid variants play a greater role in mate choice than nucleotides is easily explainable as peptides directly form phenotypic traits recognisable by individuals. The discrepancy between randomisation results and rank-based analysis can be explained as a methodological failure in the case of the grey partridge, as the condition of free mating between each female and each male is unfulfilled in a genetically monogamous species with a high mortality rate, leading to an inaccurate definition of the confidence interval for random pairs.

Both absolute and relative mate choice criteria can theoretically be combined within the same species (e.g., [25, 32, 35]). Females, for example, might choose complementary genes among the subset of males with the best genes, or they might select the most vigorous males among those showing similar levels of complementarity [25]. Among grey partridges, males with redder carotenoid ornament were more likely to obtain females, implying that absolute mate-choice criteria (attractiveness) may play some role in phenotype-based mating decisions. Interestingly, previous studies have found that phenotypic traits such as breast-patch size or periorbital hue are less important in grey partridge mate choice than behavioural differences such as vigilance or calling activity [51, 52] (not measured in this study). Nevertheless, expression of condition-dependent carotenoid ornament [50] was unaffected by any of the variants examined at MHCIIB exon 2. Likewise, presence of any of the MHCIIB variants proved unimportant for male pairing success. Thus, our results do not support the idea that the phenotypic trait preferred by grey partridge females is associated with MHC alleles determining mate choice. Similarly, the individual number of MHCIIB variants also proved unimportant for pairing success (though the MHC region investigated consisted of only two loci, hence variation in this genetic parameter was low at two to four alleles per individual). Galliformes in general appear to have a low number of genes at MHCIIB loci, in contrast to passerines [53]. Taken together, our results point to the relative unimportance of the ‘good genes’ and/or ‘heterozygosity as good genes’ hypotheses in grey partridge mate choice, at least insofar as MHCIIB exon 2 is concerned.

On the other hand, disassortative mating in grey partridges based on shared amino acid variants, as confirmed in our study, provides one of the first pieces of evidence to date for MHC-based mate choice for complementarity in a bird species (but see also [25, 29, 54]). It is nevertheless important to note that our study was limited only to MHCIIB exon 2, and that studies using more loci (e.g., both MHC classes I and II) have provided stronger support for pairing related to MHC dissimilarity [55].

It has been suggested [12] that mate choice based on genetic complementarity is more likely to evolve in long-lived species engaged in lifelong monogamy. Although grey partridges are short-lived birds with a usual lifespan of less than 1 year [48, 56], mate choice in just one season should nevertheless be of great importance as it affects lifetime reproductive success of most individuals in the population. Strong selection pressure should also act on social mate choice optimisation in this short-lived species, therefore, and especially in a pairing system with no extra-pair young [43].

A general preference for MHC-dissimilar partners enhances MHC diversity in the young, thereby improving their ability to cope with pathogens (‘heterozygote advantage principle’) [12, 31]; however, increased offspring immunocompetence may also be achieved by optimising MHC diversity in young at an intermediate level [16]. It is noteworthy that studies concerning mate choice and MHC genes have provided inconsistent results across species and different measures of genetic similarity (reviewed in [55]). Several studies have reported that individuals prefer partners with intermediate MHC dissimilarity rather than those with high dissimilarity (e.g., [27]), and that individuals may even tend to pair with mates with whom they share more MHC alleles [26]. In agreement with our results, however, several studies across taxa have documented a trend to maximise dissimilarity at MHC loci between partners in pairs [16], or an increased reproductive investment in one sex paired with a more MHC-dissimilar mate [54, 57].

Although MHC-based mating may evolve to produce offspring more resistant to pathogens, we can hypothesise that, in grey partridges, individual MHC variants are used (independently of their function in antigen binding) to distinguish genetically related animals and to avoid mating with them in order to prevent inbreeding depression in progeny [58–60]. Such inbreeding avoidance with respect to MHC may be of particular importance in species endangered by inbreeding depression (e.g., [61–65]) such as the sedentary grey partridge, whose population size has decreased dramatically throughout Europe in recent years [66]. In other words, the observed MHC-based mate choice pattern may have arisen as a by-product of general inbreeding avoidance between closely related individuals. Further evaluation of this hypothesis would require additional testing to show whether similarity at MHCIIB exon 2 is a real indicator of overall genetic relatedness between individuals, confirmed through genome-wide sequence comparisons or analyses of neutral markers [67]. A simple calculation of relatedness based on seven microsatellites and 10 individuals sampled from the studied population in 2009, whose genotypes were determined for purposes of a paternity study [43], did not show any closely related individuals in the analysed sample (Rymešová unpubl.). Unfortunately, this subset represents only 10% of individuals with known MHCIIB genotypes.

Preferential mating with genetically dissimilar individuals assumes that (1) combinations of different MHC variants affect individual phenotypes, especially as regards odour (reviewed in [21, 68]), or that (2) individuals are able to recognise both their own genotype and that of potential partners. Such a complex set of traits has been suggested as evolving mainly in mammals, reptiles and fish ([16], see [69] for a possible experimental design to test this assumption), i.e., in taxa with well-developed olfaction. Although most bird species are considered microsmatic, or even anosmic (but see [70–72]), Strandh et al. [29] have suggested that MHC-dissimilar partners of blue petrels (*Halobaena caerulea*) could be detected using olfaction. Furthermore, Leclaire et al. [73] showed that similarity in preen secretion chemicals is positively correlated with MHC relatedness in black-legged kittiwakes (*Rissa tridactyla*). While the role of olfaction for mate choice in grey partridges and other galliform birds remains unclear (see also [72]), previous studies have indicated that Galloanserae birds display a significant sense of olfaction [74]. In domestic chickens, for example, reduced male interest in females was induced experimentally by blocking secretion of the uropygial gland [75]. We can only speculate on whether the uropygial gland plays a role in determining individual odours in grey partridges, though the idea definitely merits further investigation. Taken together, though we found female preference for dissimilar males in the grey partridge based on allele sharing at MHCIIB loci, the direct mechanism for recognition of compatible partners remains unknown and it appears not to be linked with the phenotypic traits investigated herein.

## Conclusions

Our study provides one of the first pieces of evidence for MHC-based mate choice for genetic complementarity in a strictly monogamous bird, the grey partridge. We found no evidence for absolute mate choice criteria as regards grey partridge MHCIIB genotypes, i.e., number and occurrence of amino acid variants, but red chroma of the spot behind eyes was positively associated with male pairing success. On the other hand, mate choice at MHCIIB was based on relative criteria as females preferentially paired with more dissimilar males having a lower number of shared amino acid variants. This observation supports the ‘complementary genes’ hypothesis, but ‘inbreeding avoidance’ cannot be ruled out as a primary mechanism of the described mate choice pattern, as these two hypotheses could not be evaluated separately in our study. The statistical approach employed can be recommended for testing mating preferences in cases where availability of potential mates (recorded with an appropriate method such as radio-tracking) shows considerable temporal variation.

## Methods

### Study area and species management

The study was carried out in a hilly, agricultural landscape (altitude 490–619 m a.s.l.) near the villages of Milešín and Nová Ves (49°22′58.264′′N, 16°12′8.36′′E) in the Czech Republic. The core study area covered 17 km^2^ and consisted of arable land (79.3%), forest fragments (7.9%), smaller patches of shrubs in fields (6.0%), a few villages (3.4%), uncultivated grassy patches (2%) and small ponds or streams (1.4%). Severe winters with persistent snow cover are typical for the area (average January temperature: −2 to −3 °C; 130–140 days frost per year; 60–100 days snow cover per year [76]). As a result, species management measures are used by local gamekeepers to reduce winter losses in the grey partridge population [56]. Wild partridges have traditionally been trapped on snow at feeding sites and kept in captivity in their original coveys until the end of winter (mean time spent in captivity = 30 days, range = 21 to 39 days). This practice enabled us to measure and release all individuals caught during the winter at the same time before mate choice started.

### Sampling

In total, 111 adult (47 females, 64 males) free-living grey partridges were caught in the study area between 2009 and 2011. Most individuals were captured by gamekeepers using traps at feeding sites during January or February (2009: *n* = 49; 2010: *n* = 34; 2011: *n* = 4; Additional file 10). These partridges were placed in separate 2 × 4 × 2 m chambers preserving the original coveys and supplied with water and grain until release in early March. Before release of coveys at their capture site, all birds were ringed, aged, sexed and measured (wing length, tarsus length, weight). Around 1 ml of blood was also obtained from each individual by brachial venipuncture and stored in 96% ethanol at −20 °C until DNA extraction. Finally, each individual was equipped with a necklace radio tag (TW-4 or PIP3, Biotrack, Wareham, UK, weight = 6.5 g).

### Analysis of ornament

A digital photograph of the ornament on each bird was taken using a Perfection V10 scanner (Seiko Epson Corporation, Nagano, Japan) under standardised conditions (using the same position for each bird and a cm scale bar). We then documented the area of the horseshoe-shaped patch on the breast and the area and colouration components of the red spot formed by bare skin behind the eye. The size of both ornaments were measured on each standardised photograph using Adobe Photoshop CS3 v. 10.0 (Adobe Systems Inc., San Jose, CA, USA). Red spot colouration components were analysed with an Avaspec 2048 spectrometer using an Avalight XE light source (Avantes, Erbeek, Netherlands). A reflection probe (2 mm diameter) was placed perpendicularly at three points on the ornament on both sides of the head. Two variables describing red colouration were calculated from the spectral measurements according to Mougeot et al. [77]: (1) red chroma (percent reflectance between 600 and 700 nm, relative to total brightness), and (2) UV chroma (percent reflectance between 300 and 400 nm, relative to total brightness; more details in [50]). Since red chroma was strongly correlated with UV chroma (*r* = −0.886, *n* = 45 males, *p* < 0.05), only red chroma (hereinafter “redness”) was used for further analysis.

### Radio-tracking

After pair formation, both partners always stayed close together, enabling easy identification of marked individuals in pairs. If a radio-tagged bird was observed in a pair with an unmarked mate, an additional nighttime capture of the pair was conducted using a strong spot light and hand-held net in March or early April. In total, 24 individuals (12 females, 12 males; Additional file 10) were additionally captured using this method, each bird being processed immediately and rereleased at the capture site. This method also served to recapture seven individuals surviving in the study area for more than 12 months (the battery lifetime of the radio-tag).

Radio-tracking, using a hand-held Yagi antenna and a portable receiver (Sika, Biotrack, Wareham, UK; AR8000, AOR, Tokyo, Japan; Ic-R10, Icom, Osaka, Japan), was carried out daily from release of the tagged partridges until mid-May (i.e., the beginning of incubation) in order to record all potential mates of an individual. Over this period, at least one fix was obtained per day for each bird. Thereafter, marked individuals were checked three times per week until covey formation in August, and once per week until the next covey break-up in spring. Individual locations were obtained through successive triangulation or the homing-in method [78] and marked on printed orthophoto maps. Additional data (date, time, locality, individual codes of radio-tagged birds, group size, habitat, observation of the individual and its behaviour) were recorded for each radio fix on a printed form. Further data analysis was performed using ArcView v. 3.2 software (Esri, Redlands, CA, USA).

The number of days from release until first pair formation in females ranged between 1 and 17 days (median = 4 days), while males formed pairs between 1 and 84 days after release (median = 7 days). Of the 76 pairs monitored, only two ‘divorced’ without the death of a partner, and only two survived until the next nesting season. Aside from seven individuals that were lost with unknown fate (Rymešová unpubl.), death of a partner was the cause of break-up in all other cases (see also Additional file 8). Such high predation rates and the monogamous mating habits of the grey partridge stress the need to take into account the inaccessibility of already paired or dead males to unpaired females. In response, we adopted an alternative (more realistic) statistical approach based on exact daily radio-tracking data (see below).

### MHC genotyping

Genomic DNA was extracted from blood using the DNeasy Blood and Tissue Kit (Qiagen, Hilden, Germany), according to the manufacturer’s instructions. In MHC class IIB, the antigen-binding part of the protein is encoded by exon 2; hence, we amplified the whole exon 2 of MHCIIB through polymerase chain reaction (PCR) using the primers BLBintr1-Fw (5′-TGC CCG CAG CGT TCT TCC TC-3′) and BLBintr2-Rev (5′-TCA CCT TGG GCT CCA CTG CG-3′), according to the conditions described in Promerová et al. [79]. Individual genotypes were obtained using capillary electrophoresis - single-strand conformation polymorphism (CE-SSCP) method with fluorescently labelled primers in an automatic sequencer (3130 Genetic Analyser, Applied Biosystems,Waltham, MA, USA [79]). All results were analysed in GeneMapper v. 3.7 (Applied Biosystems). Although we were not able to assign the exon 2 sequences to particular loci, they are referred to as alleles for simplification. Sequences of alleles detected by CE-SSCP were obtained through bacterial cloning using the CloneJetTM PCR cloning kit (Fermentas, Waltham, MA, USA) and Sanger sequencing. All alleles were cloned and Sanger sequenced from at least two independent PCR runs and, whenever possible, from at least two individuals in order to rule out possible underestimation of variation caused by SSCP. Eight individuals were not genotyped, probably due to poor DNA quality, and four individuals carried an allele that we failed to clone (Pepe-DAB*18). As a result, 99 of 111 individuals (44 females and 55 males) were included in analysis of assortative mating.

We used four measures of MHC similarity between pair mates. Genetic distances (mean pairwise p-distance between alleles based on amino acid sequences and mean nucleotide distance) were computed in MEGA v. 5.03 [80]. MHC allele sharing (i.e., twice the sum of alleles shared by a male and a female divided by the sum of all alleles of the male and the female) was taken to represent similarity of partners in a pair [81]. Similarity was computed for both amino acid variants and nucleotide alleles. Four individuals (one male, three females) in the genetic similarities dataset were missing from the genetic distance dataset.

### Statistical analysis

Body condition was assessed as the scaled mass index according to the formula described by Peig and Green [82]*.* To obtain the slope from a standardised major axis regression of ln(mass) and ln(tarsus length), we used the lmmodel2 package in R [83]. Males were considered as paired or unpaired according to their pairing status at the end of March, the point at which all females had already formed pairs. This approach reflects female preferences more closely than final pairing status, which is based on the entire nesting season and can lead to classification of formerly single males as having successfully obtained a mate only because they pair with females after the death of the original partner. These single males may be chosen by widowed females later in the nesting season and could still nest successfully, despite their initial failure to form a pair. In such cases, the success of the male is a direct consequence of the death of the originally preferred partner.

To evaluate the effect of male attributes on male pairing success (i.e., ‘absolute’ mate choice criteria), we used generalised linear models with male pairing status (0 - unpaired: *n* = 22; 1 - paired: *n* = 33) as a binary dependent variable (logit link function; [83]). First, we evaluated the effects of particular MHC alleles (15 nucleotide alleles) and amino acid variants (13 identical and one new combination in comparison with nucleotide alleles) on male pairing status using 16 univariate models. This exploratory analysis was performed in order to choose the MHC alleles or amino acid variants most likely to be associated with pairing success in grey partridges; these then being introduced into a multivariate model in case of significance. As a second step, we evaluated the effects of overall number of amino acid variants, body condition and male ornamentation (size of horseshoe patch, average size and red chroma of the red spot) on pairing status. All continuous variables were standardised using the *scale* function in R [83]. In addition, the information theoretic approach (MuMIn package) was used to conduct a best model selection procedure on the basis of AIC [83–85]. The significant predictor of pairing success from the best model was then used in the further analysis to evaluate whether it could be affected by occurrence of any MHC allele or its amino acid variant, again using the set of all possible 16 univariate models (one for each allele or unique amino acid variant [as predictor] and phenotypic trait [dependent variable] combination). Holm correction was applied to the significance level in all cases of multiple testing.

A customised R script was used for (dis-)assortative mating assessment on the basis of pairwise amino acid distances and nucleotide distances and similarities according to sharing of amino acid variants and nucleotide alleles (‘self-referential’ mate choice criteria). The *test.pairing* function repeatedly defined random pairs (their count corresponding to the number of pairs with known genetic characteristics in a given year; 10 pairs in 2009, 21 pairs in 2010, 5 pairs in 2011) and calculated the 95% confidence interval for the median of the chosen measure of (dis-)similarity between partners from all possible male and female pairs in a given year. This was then compared with the median (dis-)similarity value based on actual pairing. If the compared value of similarity or genetic distance obtained from actual pairs fell outside the computed confidence interval for random pairs, the hypothesis of random pairing in this trait was rejected at the level *p* < 0.05. Although the morphological characteristics of both partners were obtained for 15 pairs sampled in 2009, complete MHCIIB data were only available for 10 of these pairs. Random mating was tested on a sample of 28 males and 22 females in 2009 (2 females paired twice), 28 males and 21 females in 2010 (including five males surviving from 2009, two males and three females paired twice), and 12 males and seven females in 2011 (including three females surviving from 2010, six males from 2010 and one male from 2009). Repeated mate choice was regarded as an independent pairing in this analysis.

An alternative approach for (dis-)assortative mating assessment took into account the high predation rate and monogamous mating of grey partridges and worked only with actual unpaired mates on the basis of their individually defined pairing periods. A list of candidate males was prepared for each female and her first pairing period with a known male. In such cases, the number of males available to each female differed. All unpaired males alive on the day when the female was observed in a pair for the first time were regarded as accessible candidates. These possible candidates were ranked according to the variable tested (sharing of amino acid variants in the pair, sharing of alleles, pairwise amino acid distance, and nucleotide distance). The key for ranking was prepared on the basis of the ‘complementary genes’ hypothesis, which suggests that a female will prefer the male with lowest similarity or with greatest genetic distance compared to her own MHC genotype. Each list of candidates was scored from 1 (the best candidate) to *n* (representing the worst candidate), where *n* was the female-specific number of potential mates with unique values of a genetic similarity measure. Finally, a generalised linear model assuming over-dispersion of the response variable was created in R v. 2.12.2 [83, 84] (glm.x < − glm (cbind (rank-1, num - rank) ~ +1, family = quasibinomial), where “rank” is a variable containing the rank of the chosen partner and “num” is the number of all accessible candidates with unique values for each female. Thus, the dependent variable was the number of males that should have been preferred over the actually chosen male according to the compatibility hypothesis versus the number of males that were less attractive than the chosen male. Each female was represented only once in the dataset. We tested the hypothesis that the rank of chosen males was less than *n*/2 (i.e., that females prefer males toward the top of the ranked list) and that the intercept of the model is less than zero. As this is clearly an asymmetric hypothesis, the estimated probabilities of the difference from zero were divided by two when the intercept value was negative. Finally, Holm correction was applied as we tested four measures of genetic similarity (pairwise amino acid distance and nucleotide distance, similarity on the basis of shared amino acid variants, and allele-sharing similarity) in separate models. All statistical analyses were carried out in R v. 2.12.2 [83, 84] and Statistica v. 10 (StatSoft Inc., Tulsa, OK, USA).

## Additional files

Additional file 1: MHCIIB allele frequencies in males (*n* = 56) and females (*n* = 47) in a free-living grey partridge population. (DOC 54 kb)
Additional file 2: Results of exploratory analysis showing the effect of MHCIIB variants on male grey partridge pairing status (*n* = 55). (DOC 49 kb)
Additional file 3: MHCIIB allele occurrence in paired (*n* = 35) and unpaired (*n* = 21) grey partridge males from a free-living population (2009–2011). (DOC 57 kb)
Additional file 4: The best model selection results explaining the pairing status of grey partridge males (*n* = 41). (DOC 71 kb)
Additional file 5: Descriptive statistics of morphological characteristics in paired and unpaired grey partridge males. (DOC 34 kb)
Additional file 6: Effect of MHCIIB variants on carotenoid-based ornament redness in grey partridge males (*n* = 41). (DOC 50 kb)
Additional file 7: Randomisation testing results of (dis-)assortative mating in grey partridges using nucleotide variables. (DOC 26 kb)
Additional file 8: Sex-specific monthly mortality rate of radio-tracked grey partridges in the study area (2009 to 2011 combined). (DOC 47 kb)
Additional file 9: Results of generalised linear models assessing (dis-)assortative mating in grey partridges (rank-based approach with nucleotide variables). (DOC 29 kb)
Additional file 10: Number of radio-tagged grey partridges observed between 2009 and 2011. (DOC 30 kb)

## Abbreviations

AIC: Akaike information criterion
CE: Capillary electrophoresis
glm: Generalised linear model
MHC: Major histocompatibility complex
MHCIIB: Major histocompatibility complex class IIB
SSCP: Single-strand conformation polymorphism
