# Supplementary material for: Mate choice for major histocompatibility complex complementarity in a strictly monogamous bird, the grey partridge (Perdix perdix)
Source: Front Zool. 2017 Feb 16;14:9. doi: 10.1186/s12983-017-0194-0 (PMC5312559; doi:10.1186/s12983-017-0194-0)
Supplement: Additional file 1: — MHCIIB allele frequencies in males (n = 56) and females (n = 47) in a free-living grey partridge population. (DOC 54 kb) [file 12983_2017_194_MOESM1_ESM.doc]

| **Additional file 1** |
| --- |
| **MHCIIB allele frequencies in males (*n* = 56) and females (*n* = 47) in a free-living grey partridge population.** (Alleles no. 05, 10, 11 – see Promerová et al. 2013 [79]).  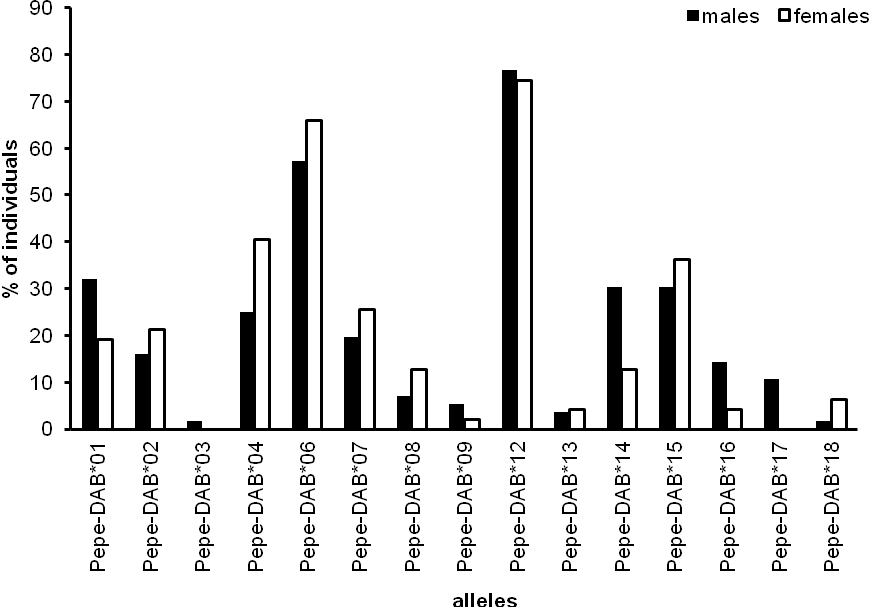 |
