# Supplementary material for: Mate choice for major histocompatibility complex complementarity in a strictly monogamous bird, the grey partridge (Perdix perdix)
Source: Front Zool. 2017 Feb 16;14:9. doi: 10.1186/s12983-017-0194-0 (PMC5312559; doi:10.1186/s12983-017-0194-0)
Supplement: Additional file 2: — Results of exploratory analysis showing the effect of MHCIIB variants on male grey partridge pairing status (n = 55). (DOC 49 kb) [file 12983_2017_194_MOESM2_ESM.doc]

| **Additional file 2** |  |  |  |
| --- | --- | --- | --- |
| **Results of exploratory analysis showing the effect of MHCIIB variants on male grey partridge pairing status (*n* = 55).** Owing to multiple testing  p values were adjusted using Holm correction (p adj). Stated values are identical for 13 nucleotide alleles (coded with „A“ in the second column) and 13 amino acid variants (coded with „P“ as products), but two alleles ( Pepe-DAB*12, Pepe-DAB*14) coded the same product, which is marked separately as Pepe-DAB*12+14 in this table.   | **Predictor** | **Allele / product** | **Estimate** | **SE** | **z value** | ***p*** | **AIC** | | ***p adj*** | | | --- | --- | --- | --- | --- | --- | --- | --- | --- | --- | | Pepe-DAB*01 | A, P | 0.793 | 0.621 | 1.277 | 0.202 | 76.320 | 1.000 | |  | | Pepe-DAB*02 | A, P | -0.219 | 0.736 | -0.297 | 0.766 | 77.943 | 1.000 | |  | | Pepe-DAB*03 | A, P | -16.018 | 1455.398 | -0.011 | 0.991 | 76.171 | 1.000 | |  | | Pepe-DAB*04 | A, P | -0.331 | 0.641 | -0.517 | 0.605 | 77.765 | 1.000 | |  | | Pepe-DAB*06 | A, P | -0.823 | 0.575 | -1.430 | 0.153 | 75.917 | 1.000 | |  | | Pepe-DAB*07 | A, P | 1.322 | 0.838 | 1.577 | 0.115 | 75.064 | 1.000 | |  | | Pepe-DAB*08 | A, P | 17.290 | 1978.090 | 0.009 | 0.993 | 73.737 | 1.000 | |  | | Pepe-DAB*09 | A, P | -1.163 | 1.258 | -0.925 | 0.355 | 77.112 | 1.000 | |  | | Pepe-DAB*12 | A | -0.365 | 0.686 | -0.532 | 0.595 | 77.742 | 1.000 | |  | | Pepe-DAB*13 | A, P | -0.421 | 1.442 | -0.292 | 0.770 | 77.946 | 1.000 | |  | | Pepe-DAB*14 | A | -0.219 | 0.602 | -0.363 | 0.716 | 77.900 | 1.000 | |  | | Pepe-DAB*15 | A, P | -0.071 | 0.594 | -0.119 | 0.905 | 78.017 | 1.000 | |  | | Pepe-DAB*16 | A, P | 0.799 | 0.868 | 0.920 | 0.358 | 77.107 | 1.000 | |  | | Pepe-DAB*17 | A, P | 1.322 | 1.133 | 1.167 | 0.243 | 76.332 | 1.000 | |  | | Pepe-DAB*18 | A, P | -16.018 | 1455.398 | -0.011 | 0.991 | 76.171 | 1.000 | |  | | Pepe-DAB*12+14 | P | -1.540 | 1.119 | -1.377 | 0.169 | 75.532 | 1.000 | |  | | | | |
