# Supplementary material for: Mate choice for major histocompatibility complex complementarity in a strictly monogamous bird, the grey partridge (Perdix perdix)
Source: Front Zool. 2017 Feb 16;14:9. doi: 10.1186/s12983-017-0194-0 (PMC5312559; doi:10.1186/s12983-017-0194-0)
Supplement: Additional file 3: — MHCIIB allele occurrence in paired (n = 35) and unpaired (n = 21) grey partridge males from a free-living population (2009–2011). (DOC 57 kb) [file 12983_2017_194_MOESM3_ESM.doc]

| **Additional file 3** |
| --- |
| **MHCIIB allele occurrence in paired (*n* = 35) and unpaired (*n* = 21) grey partridge males from a free-living population (2009–2011).** Males surviving more than one season are included only in the first year of observation. (Alleles no. 05, 10, 11 – see Promerová et al. 2013 [79]).  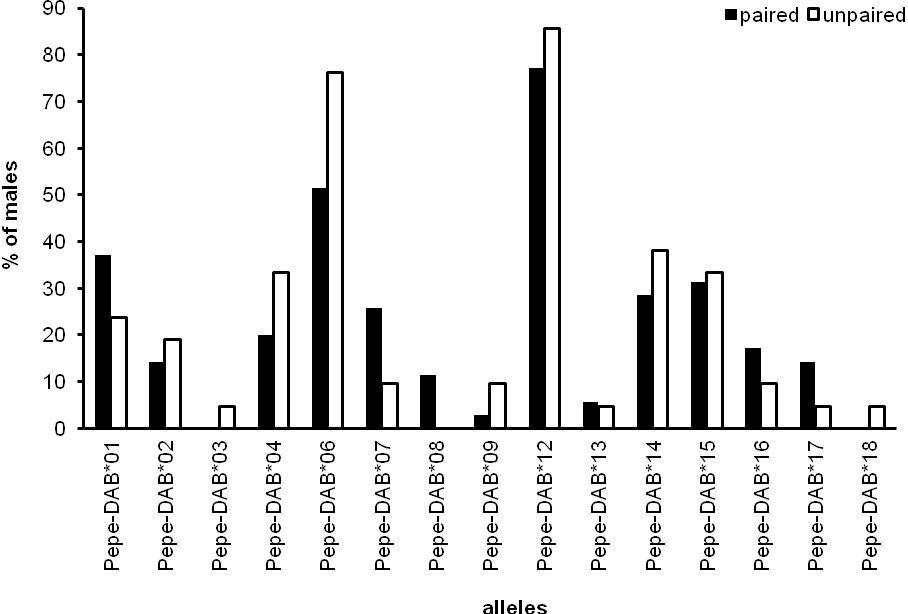 |
