# Supplementary material for: Mate choice for major histocompatibility complex complementarity in a strictly monogamous bird, the grey partridge (Perdix perdix)
Source: Front Zool. 2017 Feb 16;14:9. doi: 10.1186/s12983-017-0194-0 (PMC5312559; doi:10.1186/s12983-017-0194-0)
Supplement: Additional file 4: — The best model selection results explaining the pairing status of grey partridge males (n = 41). (DOC 71 kb) [file 12983_2017_194_MOESM4_ESM.doc]

| **Additional file 4**  **The best model selection results of explaining the pairing status of grey partridge males (*n* = 41). Abbreviations: No. – model number, Variants – number of amino acid variants per male, Redness – red chroma of the carotenoid ornament (e.g. red spot behind eyes), Spot area – mean area of the carotenoid ornament, Horseshoe – area of the horseshoe patch, Condition – scaled body condition, df - number of model parameters, logLik - Log-likelihood , AICc - second-order Akaike information criterion, weight - Akaike weights.** |
| --- |
| | No. | Intercept | Variants | Redness | Spot area | Horseshoe | Condition | df | logLik | AICc | delta | weight | | --- | --- | --- | --- | --- | --- | --- | --- | --- | --- | --- | --- | | 3 | -0.0645 |  | 0.8856 |  |  |  | 2 | -25.442 | 55.2 | 0.00 | 0.211 | | 7 | -0.0776 |  | 0.7981 | 0.4392 |  |  | 3 | -24.749 | 56.1 | 0.95 | 0.132 | | 19 | -0.0622 |  | 0.8835 |  |  | 0.2281 | 3 | -25.22 | 57.1 | 1.89 | 0.082 | | 11 | -0.0614 |  | 0.9245 |  | 0.1406 |  | 3 | -25.366 | 57.4 | 2.18 | 0.071 | | 4 | -0.0646 | -0.0376 | 0.8862 |  |  |  | 3 | -25.436 | 57.5 | 2.32 | 0.066 | | 15 | -0.0718 |  | 0.8553 | 0.5051 | 0.2562 |  | 4 | -24.513 | 58.1 | 2.94 | 0.049 | | 5 | -0.0557 |  |  | 0.5756 |  |  | 2 | -26.916 | 58.1 | 2.95 | 0.048 | | 23 | -0.0766 |  | 0.8036 | 0.4025 |  | 0.0948 | 4 | -24.716 | 58.5 | 3.34 | 0.040 | | 8 | -0.0776 | -0.0177 | 0.7980 | 0.4380 |  |  | 4 | -24.748 | 58.6 | 3.41 | 0.038 | | 1 | -0.0488 |  |  |  |  |  | 1 | -28.407 | 58.9 | 3.72 | 0.033 | | 27 | -0.0608 |  | 0.9108 |  | 0.1029 | 0.2114 | 4 | -25.181 | 59.5 | 4.27 | 0.025 | | 20 | -0.0622 | -0.0009 | 0.8835 |  |  | 0.2279 | 4 | -25.22 | 59.6 | 4.35 | 0.024 | | 12 | -0.0614 | -0.0200 | 0.9240 |  | 0.1375 |  | 4 | -25.365 | 59.8 | 4.64 | 0.021 | | 21 | -0.0565 |  |  | 0.5570 |  | 0.0523 | 3 | -26.905 | 60.5 | 5.26 | 0.015 | | 13 | -0.0561 |  |  | 0.5852 | 0.0325 |  | 3 | -26.912 | 60.5 | 5.27 | 0.015 | | 6 | -0.0557 | 0.0085 |  | 0.5758 |  |  | 3 | -26.916 | 60.5 | 5.28 | 0.015 | | 17 | -0.0490 |  |  |  |  | 0.2384 | 2 | -28.127 | 60.6 | 5.37 | 0.014 | | 31 | -0.0719 |  | 0.8550 | 0.4913 | 0.2482 | 0.0313 | 5 | -24.51 | 60.7 | 5.53 | 0.013 | | 16 | -0.0718 | 0.0186 | 0.8561 | 0.5072 | 0.2593 |  | 5 | -24.511 | 60.7 | 5.54 | 0.013 | | 9 | -0.0490 |  |  |  | -0.1333 |  | 2 | -28.319 | 61 | 5.75 | 0.012 | | 2 | -0.0488 | 0.0066 |  |  |  |  | 2 | -28.407 | 61.1 | 5.93 | 0.011 | | 24 | -0.0766 | -0.0037 | 0.8035 | 0.4025 |  | 0.0941 | 5 | -24.716 | 61.1 | 5.95 | 0.011 | | 28 | -0.0610 | 0.0096 | 0.9111 |  | 0.1040 | 0.2126 | 5 | -25.181 | 62.1 | 6.88 | 0.007 | | 25 | -0.0495 |  |  |  | -0.1639 | 0.2580 | 3 | -27.996 | 62.6 | 7.44 | 0.005 | | 18 | -0.0491 | 0.0363 |  |  |  | 0.2428 | 3 | -28.12 | 62.9 | 7.69 | 0.005 | | 29 | -0.0566 |  |  | 0.5653 | 0.0214 | 0.0471 | 4 | -26.903 | 62.9 | 7.72 | 0.004 | | 22 | -0.0565 | 0.0155 |  | 0.5566 |  | 0.0546 | 4 | -26.904 | 62.9 | 7.72 | 0.004 | | 14 | -0.0561 | 0.0151 |  | 0.5865 | 0.0356 |  | 4 | -26.911 | 62.9 | 7.73 | 0.004 | | 10 | -0.0490 | -0.0178 |  |  | -0.1364 |  | 3 | -28.317 | 63.3 | 8.08 | 0.004 | | 32 | -0.0720 | 0.0223 | 0.8559 | 0.4925 | 0.2512 | 0.0342 | 6 | -24.507 | 63.5 | 8.29 | 0.003 | | 26 | -0.0496 | 0.0089 |  |  | -0.1624 | 0.2589 | 4 | -27.996 | 65.1 | 9.90 | 0.001 | | 30 | -0.0566 | 0.0194 |  | 0.5660 | 0.0248 | 0.0491 | 5 | -26.901 | 65.5 | 10.32 | 0.001 | |
