# Supplementary material for: Mate choice for major histocompatibility complex complementarity in a strictly monogamous bird, the grey partridge (Perdix perdix)
Source: Front Zool. 2017 Feb 16;14:9. doi: 10.1186/s12983-017-0194-0 (PMC5312559; doi:10.1186/s12983-017-0194-0)
Supplement: Additional file 5: — Descriptive statistics of morphological characteristics in paired and unpaired grey partridge males. (DOC 34 kb) [file 12983_2017_194_MOESM5_ESM.doc]

| **Additional file 5** | | | | | | | |
| --- | --- | --- | --- | --- | --- | --- | --- |
| **Descriptive statistics of morphological characteristics in paired and unpaired grey partridge males.** (Pairing status was assessed until the end of March. Males surviving more than one year are included only in the first breeding season). | | | | | | | |
| **Variable** | **Unpaired males** | | | **Paired males** | | | |
|  | ***n*** | **Mean** | **SE** | ***n*** | **Mean** | | **SE** |
| Scaled body condition | 22 | 369.80 | 25.87 | 31 | 385.70 | 23.75 | |
| Weight (g) | 22 | 361.38 | 6.84 | 30 | 390.91 | 4.49 | |
| Wing length – mean (mm) | 22 | 155.07 | 0.63 | 32 | 155.36 | 0.57 | |
| Tarsus length – mean (mm) | 22 | 50.21 | 0.34 | 33 | 50.80 | 0.23 | |
| Horseshoe area (mm^2^) | 22 | 1683.86 | 98.07 | 32 | 1762.20 | 82.61 | |
| Red spot area – mean (mm^2^) | 22 | 38.43 | 2.42 | 28 | 45.13 | 2.25 | |
| Red spot chroma (%) | 21 | 50.23 | 1.5 | 21 | 55.30 | 1.18 | |
