# Supplementary material for: Mate choice for major histocompatibility complex complementarity in a strictly monogamous bird, the grey partridge (Perdix perdix)
Source: Front Zool. 2017 Feb 16;14:9. doi: 10.1186/s12983-017-0194-0 (PMC5312559; doi:10.1186/s12983-017-0194-0)
Supplement: Additional file 6: — Effect of MHCIIB variants on carotenoid-based ornament redness in grey partridge males (n = 41). (DOC 50 kb) [file 12983_2017_194_MOESM6_ESM.doc]

| **Additional file 6** |  |  |  |  |
| --- | --- | --- | --- | --- |
| **Effect of MHCIIB variants on carotenoid-based ornament redness in grey partridge males (n = 41). Owing to multiple testing  p values were adjusted using Holm correction (*p adj*). Stated values are identical for 13 nucleotide alleles (coded with „A“ in the second column) and 13 amino acid variants (coded with „P“ as products), but two alleles ( Pepe-DAB*12, Pepe-DAB*14) coded the same product, which is marked separately as Pepe-DAB*12 +14 in this table.**   | **Predictor** | **Allele / product** | **Estimate** | **SE** | ***z* value** | ***p*** | **AIC** | ***p adj*** | | --- | --- | --- | --- | --- | --- | --- | --- | | Allele Pepe-DAB*01 | A, P | 17.199 | 5345.908 | 0.003 | 0.997 | 12.77 | 1.000 | | Allele Pepe-DAB*02 | A, P | 17.132 | 5910.123 | 0.003 | 0.998 | 12.90 | 1.000 | | Allele Pepe-DAB*03 | A, P | 13.903 | 3956.180 | 0.004 | 0.997 | 13.35 | 1.000 | | Allele Pepe-DAB*04 | A, P | 17.199 | 5345.908 | 0.003 | 0.997 | 12.77 | 1.000 | | Allele Pepe-DAB*06 | A, P | -17.430 | 4300.250 | -0.004 | 0.997 | 12.31 | 1.000 | | Allele Pepe-DAB*07 | A, P | 17.132 | 5910.123 | 0.003 | 0.998 | 12.90 | 1.000 | | Allele Pepe-DAB*08 | A, P | 15.983 | 5377.007 | 0.003 | 0.998 | 13.20 | 1.000 | | Allele Pepe-DAB*09 | A, P | 14.955 | 3765.847 | 0.004 | 0.997 | 13.25 | 1.000 | | Allele Pepe-DAB*12 | A | -17.200 | 5345.910 | -0.003 | 0.997 | 12.77 | 1.000 | | Allele Pepe-DAB*13 | A, P | 13.903 | 3956.180 | 0.004 | 0.997 | 13.35 | 1.000 | | Allele Pepe-DAB*14 | A | 17.234 | 5118.317 | 0.003 | 0.997 | 12.70 | 1.000 | | Allele Pepe-DAB*15 | A, P | 17.165 | 5606.835 | 0.003 | 0.998 | 12.84 | 1.000 | | Allele Pepe-DAB*16 | A, P | 16.011 | 4809.341 | 0.003 | 0.997 | 13.14 | 1.000 | | Allele Pepe-DAB*17 | A, P | 14.955 | 3765.847 | 0.004 | 0.997 | 13.25 | 1.000 | | Allele Pepe-DAB*18 | A, P | 13.903 | 3956.180 | 0.004 | 0.997 | 13.35 | 1.000 | | Allele Pepe-DAB*12+14 | P | -16.040 | 4390.310 | -0.004 | 0.997 | 13.08 | 1.000 | | | | | |
