# Supplementary material for: Mate choice for major histocompatibility complex complementarity in a strictly monogamous bird, the grey partridge (Perdix perdix)
Source: Front Zool. 2017 Feb 16;14:9. doi: 10.1186/s12983-017-0194-0 (PMC5312559; doi:10.1186/s12983-017-0194-0)
Supplement: Additional file 7: — Randomisation testing results of (dis-)assortative mating in grey partridges using nucleotide variables. (DOC 26 kb) [file 12983_2017_194_MOESM7_ESM.doc]

**Additional file 7**

**Randomisation testing results of (dis-)assortative mating in grey partridges using nucleotide variables.** Median for real pairs was compared with limits of the 95 % confidence interval for randomly chosen pairs (based on 10000 permutations and 36 known real pairs).

| **Variable** | **95 % CI min** | **95 % CI max** | **Median** | | |
| --- | --- | --- | --- | --- | --- |
| Allele-sharing similarity | 0.2857 | 0.5714 | | 0.3333 |  |
| Mean nucleotide distance | 0.0792 | 0.0903 | | 0.0902 |  |
