# Supplementary material for: Mate choice for major histocompatibility complex complementarity in a strictly monogamous bird, the grey partridge (Perdix perdix)
Source: Front Zool. 2017 Feb 16;14:9. doi: 10.1186/s12983-017-0194-0 (PMC5312559; doi:10.1186/s12983-017-0194-0)
Supplement: Additional file 8: — Sex-specific monthly mortality rate of radio-tracked grey partridges in the study area (2009 to 2011 combined). (DOC 47 kb) [file 12983_2017_194_MOESM8_ESM.doc]

**Additional file 8.** **Sex-specific monthly mortality rate of radio-tracked grey partridges in the study area (2009 to 2011 combined).**

| Number of / Month | Mar. | Apr. | May | June | July | Aug. | Sept. | Oct. | Nov. | Dec. | Jan. | Feb. |
| --- | --- | --- | --- | --- | --- | --- | --- | --- | --- | --- | --- | --- |
| Females alive ^1^ | 49 | 37 | 33 | 24 | 15 | 12 | 9 | 8 | 7 | 6 | 5 | 4 |
| Females dead ^2^ | 9 | 3 | 9 | 9 | 3 | 2 | 1 | 1 | 0 | 1 | 0 | 0 |
| Females dead caught by a predator | 8 | 3 | 9 | 6 | 3 | 2 | 1 | 1 | 0 | 1 | 0 | 0 |
| Females lost ^3^ | 3 | 1 | 0 | 0 | 0 | 1 | 0 | 0 | 1 | 0 | 1 | 4* |
| Males alive ^1^ | 76 | 60 | 46 | 35 | 25 | 25 | 21 | 21 | 19 | 19 | 16 | 14 |
| Males dead ^2^ | 11 | 12 | 7 | 8 | 0 | 4 | 0 | 2 | 0 | 3 | 2 | 0 |
| Males dead caught by a predator | 9 | 10 | 7 | 6 | 0 | 4 | 0 | 2 | 0 | 2 | 1 | 0 |
| Males lost ^3^ | 5 | 2 | 4 | 2 | 0 | 0 | 0 | 0 | 0 | 0 | 0 | 14* |
| Mortality rate (%) / Month | Mar. | Apr. | May | June | July | Aug. | Sept. | Oct. | Nov. | Dec. | Jan. | Feb. |
| Relative - females ^4^ | 18.4 | 8.1 | 27.3 | 37.5 | 20.0 | 16.7 | 11.1 | 12.5 | 0.0 | 16.7 | 0.0 | 0.0 |
| Lost females count as dead ^5^ | 24.5 | 10.8 | 27.3 | 37.5 | 20.0 | 25.0 | 11.1 | 12.5 | 14.3 | 16.7 | 20.0 |  |
| Absolute - females ^6^ | 18.4 | 6.1 | 18.4 | 18.4 | 6.1 | 4.1 | 2.0 | 2.0 | 0.0 | 2.0 | 0.0 | 0.0 |
| Relative - males ^4^ | 14.5 | 20.0 | 15.2 | 22.9 | 0.0 | 16.0 | 0.0 | 9.5 | 0.0 | 15.8 | 12.5 | 0.0 |
| Lost males count as dead ^5^ | 21.1 | 23.3 | 23.9 | 28.6 | 0.0 | 16.0 | 0.0 | 9.5 | 0.0 | 15.8 | 12.5 |  |
| Absolute - males ^6^ | 14.5 | 15.8 | 9.2 | 10.5 | 0.0 | 5.3 | 0.0 | 2.6 | 0.0 | 3.9 | 2.6 | 0.0 |

* Number of individuals survived until the end of observation, i.e. one year, which was the limit of the battery in radio-tags.

^1^ Number of females/males being alive at the beginning of the month.

^2^ Numbers of females/males found dead during the month (including mostly predation rarely illnesses or human-caused cases).

^3^ Loss of the radio-tag signal, which can be caused by a technical failure, a damage of radio-tag by a predator or an extensive dispersion of the individual.

^4^ Proportion of dead females/males in the month from the number of females/males alive at the beginning of the month (%).

^5^ Proportion of dead and lost females/males in the month from the number of females/males alive at the beginning of the month (%).

^6^ Proportion of dead females/males in the month from the number of originally released females/males alive at the beginning of March (%).
