# Supplementary material for: Mate choice for major histocompatibility complex complementarity in a strictly monogamous bird, the grey partridge (Perdix perdix)
Source: Front Zool. 2017 Feb 16;14:9. doi: 10.1186/s12983-017-0194-0 (PMC5312559; doi:10.1186/s12983-017-0194-0)
Supplement: Additional file 9: — Results of generalised linear models assessing (dis-)assortative mating in grey partridges (rank-based approach with nucleotide variables). (DOC 29 kb) [file 12983_2017_194_MOESM9_ESM.doc]

**Additional file 9**

**Results of generalised linear models assessing (dis-)assortative mating in grey partridges (rank-based approach with nucleotide variables). Relative ranks of actually chosen mates from accessible candidates for each female were used (*n* = 32 females, 5 duplications were excluded). Significant values are marked in bold (*p* < 0.05). The last column contains values of probability after the Holm correction.**

| **Predictor** | **Estimate** | **SE** | **z value** | ***p*** | | ***p/2*** | **Holm** |
| --- | --- | --- | --- | --- | --- | --- | --- |
| Allele-sharing similarity | -0.7967 | 0.2325 | -3.426 | **0.0017** | **0.0009** | | **0.0034** |
| Mean nucleotide distance | -0.2364 | 0.2168 | -1.090 | 0.2840 | 0.1420 | | 0.2840 |
