# Supplementary material for: Mate choice for major histocompatibility complex complementarity in a strictly monogamous bird, the grey partridge (Perdix perdix)
Source: Front Zool. 2017 Feb 16;14:9. doi: 10.1186/s12983-017-0194-0 (PMC5312559; doi:10.1186/s12983-017-0194-0)
Supplement: Additional file 10: — Number of radio-tagged grey partridges observed between 2009 and 2011. (DOC 30 kb) [file 12983_2017_194_MOESM10_ESM.doc]

**Additional file 10**

**Number of radio-tagged grey partridges observed between 2009 and 2011.** Abbreviations: M – males, F – females.

| **Year** | **Trapped** | | | **Additionally** | | | **Surviving from previous year** | | | | | |
| --- | --- | --- | --- | --- | --- | --- | --- | --- | --- | --- | --- | --- |
|  | **in winter** | | | **captured** | | | **original radio-tags** | | | **exchanged radio-tags** | | |
|  | **M** | **F** | **All** | **M** | **F** | **All** | **M** | **F** | **All** | **M** | **F** | **All** |
| 2009 | 28 | 21 | 49 | 6 | 1 | 7 | 0 | 0 | 0 | 0 | 0 | 0 |
| 2010 | 21 | 13 | 34 | 2 | 8 | 10 | 5 | 0 | 5 | 2 | 0 | 2 |
| 2011 | 3 | 1 | 4 | 4 | 3 | 7 | 8 | 4 | 12 | 4 | 1 | 5 |
| Total | 52 | 35 | 87 | 12 | 12 | 24 | 13 | 4 | 17 | 6 | 1 | 7 |
